# Creatine Supplementation in Endurance and Mixed-Sport Contexts: A Scoping Review of Performance, Recovery, and Body Composition

**DOI:** 10.3390/nu18111677

**Published:** 2026-05-24

**Authors:** Igor Wesołowski, Jacek Dzienisiewicz, Dorota Langa, Wiesław Ziółkowski, Joanna Karbowska, Zdzislaw Kochan

**Affiliations:** 1 Laboratory of Nutritional Biochemistry, Department of Clinical Nutrition, Medical University of Gdansk, 80-211 Gdansk, Poland; igorwesolowski@gumed.edu.pl (I.W.); asmaokai@gumed.edu.pl (J.D.); dorota.langa@gumed.edu.pl (D.L.); 2 Department of Rehabilitation Medicine, Medical University of Gdansk, 80-219 Gdansk, Poland; wieslaw.ziolkowski@gumed.edu.pl; 3 Department of Biochemistry, Medical University of Gdansk, 80-211 Gdansk, Poland

**Keywords:** ergogenic aids, phosphocreatine, bioenergetics, sport nutrition, repeated-sprint ability, power output, aerobic capacity, inflammation, exercise-induced muscle damage

## Abstract

**Background/Objectives**: Although creatine monohydrate is widely recognized as an effective ergogenic aid in strength and power sports, its role in endurance and mixed-sport disciplines remains less clearly established. This scoping review aimed to map the current evidence regarding the effects of creatine supplementation on performance, recovery-related outcomes, and body composition in endurance and mixed-sport contexts. **Methods**: A scoping review of randomized controlled trials published between 1996 and 2025 was conducted. Eligible studies evaluated creatine supplementation in endurance and mixed-sport contexts, including both sport-specific and broader exercise populations when the exercise protocol, testing model, or outcomes were relevant to endurance or mixed-sport performance, recovery, or body composition. A total of 38 studies met the inclusion criteria. Outcomes were categorized into exercise performance, biochemical markers related to recovery and exercise stress, and body composition parameters. **Results**: Creatine supplementation was most often associated with reported favorable changes in repeated-sprint performance and high-intensity power output, particularly during intermittent, sprint-based, or power-endurance tasks. Several studies reported favorable changes in sprint performance, peak power, or total work output relative to placebo or baseline values in cycling, swimming, rowing, and canoeing/kayaking protocols, although findings were not uniform across studies and not all favorable within-group changes were placebo-superior. Some studies also reported favorable changes in end-phase sprint capacity during prolonged exercise. Findings related to recovery were less consistent. Selected studies reported reductions in inflammatory markers, including C-reactive protein (CRP) and tumor necrosis factor α (TNF-α), whereas markers of muscle damage showed mixed responses. Most supplementation protocols involved a 5–7-day loading phase of 20 g/day, occasionally followed by a maintenance dose of 2–5 g/day. Small increases in total body mass were commonly observed, while evidence regarding fat-free mass and aerobic outcomes remained limited or inconsistent. **Conclusions**: Current evidence suggests that creatine supplementation may be most relevant in selected endurance and mixed-sport contexts involving repeated high-intensity efforts, sprint finishes, or power-endurance demands, rather than for endurance performance broadly. In contrast, evidence for recovery-related biochemical responses, body composition changes, and aerobic adaptations remains equivocal. Further well-controlled, sport- or context-specific, and field-based studies are needed to better clarify the role of creatine in endurance and mixed-sport exercise.

## 1. Introduction

Performance in endurance and mixed-sport disciplines typically involves prolonged or repeated whole-body activity supported largely by aerobic energy production. Endurance sports encompass disciplines such as running, cycling, swimming, rowing, and cross-country skiing, with events that often last from several minutes to several hours—or even days in ultra-endurance contexts—placing sustained demands on the cardiovascular and muscular systems [1]. Mixed sports, as considered in this review, include intermittent disciplines such as team, combat, and racket sports, in which substantial aerobic demands coexist with repeated high-intensity or anaerobic efforts. Although these sports involve frequent anaerobic actions, overall performance remains strongly influenced by aerobic metabolism and recovery capacity. This distinguishes them from predominantly anaerobic disciplines, such as sprinting, throwing events, or Olympic weightlifting, which rely primarily on short-duration maximal power output and fall outside the scope of this review. At the cellular level, performance in endurance and mixed disciplines depends on efficient oxidative phosphorylation, high mitochondrial density, and sustained adenosine triphosphate (ATP) turnover, with energy derived primarily from carbohydrate and lipid oxidation [2].

While aerobic metabolism provides the foundation for performance in endurance and many mixed sports, real-world competition rarely proceeds at a steady pace. Most events include repeated high-intensity efforts—such as surges, climbs, tactical attacks, and sprint finishes—that rely on anaerobic ATP provision, particularly the ATP–phosphocreatine (PCr; phosphagen) system and anaerobic glycolysis. These bursts can rapidly deplete intramuscular energy stores, increase hydrogen ion accumulation, and accelerate fatigue, especially under competitive conditions [2,3]. Consequently, athletes in endurance and mixed disciplines require not only aerobic efficiency but also anaerobic resilience to meet the complex physiological demands of racing.

Beyond these acute race demands, prolonged training and competition impose cumulative stress on skeletal muscle, can increase oxidative damage, and contribute to delayed recovery—especially during periods of high training volume or multi-stage events [4]. In this context, nutritional strategies that support energy metabolism, cellular homeostasis, and recovery are increasingly important components of performance planning.

Among nutritional strategies examined for their potential to support these physiological demands, creatine has emerged as a supplement of considerable interest in sport science and applied practice. Creatine gained widespread attention in elite sport during the 1990s, particularly following reports of its use by Olympic athletes at the Barcelona and Atlanta Games [5,6]. Since then, creatine monohydrate has become one of the most extensively studied and widely used ergogenic supplements. Sport nutrition literature, including position stands, supports its efficacy primarily for strength, power, and short-duration performance [7]. Its role in endurance and mixed-sport contexts remains less straightforward, with potential benefits appearing more likely when performance depends on repeated surges, sprint finishes, or recovery between high-intensity bouts rather than on steady-state aerobic capacity alone [3,8]. Emerging research suggests that creatine may also offer benefits beyond its classical scope through mechanisms including increased glycogen storage, muscle cell hydration, buffering intramuscular acidosis, and attenuation of muscle damage and inflammation [3]. These adaptations may be particularly relevant in endurance and mixed-sport contexts characterized by cumulative fatigue, repeated high-intensity efforts, and high training loads.

Current evidence provides an incomplete and inconsistent picture of creatine’s role in endurance and mixed sports. While some studies report improvements in time to exhaustion, recovery, or muscle preservation, others find no meaningful benefits for sport-specific performance. The literature spans a wide range of supplementation protocols, participant populations, and exercise models, including both sport-specific athlete samples and broader exercise-based populations, with relatively few studies conducted under real-world competitive conditions.

This scoping review aims to systematically identify, map, and categorize the existing evidence on creatine supplementation in endurance and mixed-sport contexts, focusing on outcomes related to performance, recovery, and body composition. Consistent with a scoping review approach, the objective is primarily descriptive: to characterize the scope, heterogeneity, methodological limitations, and direction of the available evidence rather than to establish definitive conclusions or prescribe supplementation strategies. By identifying key findings, methodological trends, and knowledge gaps, this review may help guide future research and support cautious, evidence-informed interpretation by practitioners.

## 2. Methods

This scoping review was conducted and reported in accordance with the PRISMA-ScR (Preferred Reporting Items for Systematic Reviews and Meta-Analyses Extension for Scoping Reviews) checklist [9]. A review protocol was not registered, and no protocol was made publicly available. The scoping review design was selected to map the range, characteristics, and heterogeneity of controlled intervention evidence on creatine supplementation in endurance and mixed-sport contexts. Accordingly, eligibility was restricted to randomized controlled trials (RCTs) involving placebo or no-intervention comparators. We aimed to systematically identify and characterize RCTs examining the effects of creatine supplementation on performance, recovery, and body composition in endurance and mixed-sport contexts, as defined by the eligibility criteria. Two authors (I.W. and J.D.) independently screened titles and abstracts and assessed full-text reports for eligibility. A third author (Z.K.) cross-checked the screening decisions, eligibility assessments, and extracted data. Any disagreements were resolved through discussion.

### 2.1. Search Strategy

We searched PubMed, Scopus, Web of Science, Embase, and the Cochrane Central Register of Controlled Trials (CENTRAL) for studies published between January 1996 and April 2025. The PubMed search strategy was developed in multiple stages to maximize the relevance of the retrieved records, and equivalent search strategies were adapted for the syntax and indexing structure of each additional database. Records retrieved from all databases were imported into a reference-management file and deduplicated before screening.

In the first stage, the study population was defined using MeSH terms and keywords: (“Athletic Performance”[MeSH Terms] OR “Endurance Training”[MeSH Terms] OR athlete*[Title/Abstract] OR athletics[Title/Abstract] OR endurance[Title/Abstract] OR aerobic[Title/Abstract] OR distance[Title/Abstract] OR runner*[Title/Abstract] OR marathon*[Title/Abstract] OR cyclist*[Title/Abstract] OR triathlete*[Title/Abstract] OR triathlon[Title/Abstract] OR rower*[Title/Abstract] OR rowing[Title/Abstract] OR swimmer*[Title/Abstract] OR swimming[Title/Abstract] OR mma[Title/Abstract] OR “mixed martial arts”[Title/Abstract] OR boxing[Title/Abstract] OR “boxer*”[Title/Abstract]).

In the next stage, logical AND operators were used to restrict the results to creatine interventions. The search strategy incorporated a wide range of synonyms, brand names, and formulations related to creatine supplementation: (Creatine[Supplementary Concept] OR Creatine[MeSH Terms] OR “creatine monohydrate”[Title/Abstract] OR CrM[Title/Abstract] OR “creatine supplement*”[Title/Abstract] OR “creatine load*”[Title/Abstract] OR “creatine ethyl ester”[Title/Abstract] OR “kre-alkalyn”[Title/Abstract] OR creapure[Title/Abstract] OR Creatine*[Title/Abstract]).

To better capture outcomes related to recovery and performance, the search strategy was expanded to include specific endpoints: (strength[Title/Abstract] OR “muscle strength”[MeSH Terms] OR recovery[Title/Abstract] OR “muscle damage”[Title/Abstract] OR soreness[Title/Abstract] OR CRP[Title/Abstract] OR “serum CK”[Title/Abstract] OR “C-Reactive Protein”[Title/Abstract] OR “creatine kinase”[Title/Abstract] OR “lactate dehydrogenase”[Title/Abstract] OR LDH[Title/Abstract] OR lactate[Title/Abstract] OR “Interleukin-6”[Title/Abstract] OR “IL-6”[Title/Abstract]).

The final search was further restricted to studies involving human participants (“humans”[MeSH Terms]) and placebo (placebo[Title/Abstract]).

Similar search strategies, adapted to database-specific syntax and controlled vocabulary where applicable, were implemented in Scopus, Web of Science, Embase, and Cochrane CENTRAL. The complete database-specific search strings are provided in Supplementary Materials File S2.

### 2.2. Eligibility Criteria

The eligibility criteria were formulated according to the PICOS framework [10]. Population (P): athletes participating in endurance and mixed sports, including but not limited to swimmers, cyclists, runners, triathletes, rowers, canoeists/kayak paddlers, and athletes from intermittent team, combat, or racket sports. Intermittent team, combat, and racket sports were considered mixed-sport contexts, and not endurance disciplines, when they involved repeated high-intensity actions interspersed with lower-intensity activity or recovery periods. Studies involving broader populations, including physically active, recreationally active, not regularly training, untrained, or adaptive-sport participants, were also considered eligible when the exercise protocol, testing model, or outcome measures were relevant to endurance or mixed-sport performance, recovery, or body composition outcomes. Intervention (I): creatine monohydrate supplementation, administered alone or in combination with other ergogenic agents, such as carbohydrates or sodium bicarbonate. Comparator (C): placebo or no intervention. Outcomes (O): aerobic and anaerobic performance-related outcomes, biochemical markers related to recovery, such as TNF-α, CRP, or CK, and body composition outcomes, such as TBM or FFM. Study design (S): randomized controlled trials (RCTs) using a parallel-group or crossover design.

Studies limited to resistance training or to predominantly anaerobic sport disciplines with minimal aerobic demand were excluded. Eligible studies evaluated creatine monohydrate supplementation, either alone or in combination with another supplement, and reported at least one of the following outcomes: performance in endurance or mixed-sport contexts, recovery-related outcomes, including inflammatory or muscle-damage markers, or body composition. The inclusion of some broader or less sport-specific populations is therefore acknowledged as a source of heterogeneity in the available evidence base, and findings from these studies were interpreted cautiously rather than generalized directly to endurance or mixed-sport athletes.

### 2.3. Data Extraction

The following data were extracted from each eligible study: study design, sample size, participant characteristics, including gender distribution and training status where reported, supplementation protocol, including dose and duration, sport or exercise context, and primary outcomes. Two authors (I.W. and J.D.) independently extracted data, and a third author (Z.K.) cross-checked all extracted data for accuracy. Disagreements were resolved through discussion.

### 2.4. Methodological Characteristics

In keeping with the descriptive purpose of this scoping review, no formal risk-of-bias tool was used, and studies were not excluded or weighted based on methodological quality. Instead, methodological characteristics relevant to interpretation were descriptively charted. For each included study, we summarized randomization, blinding, allocation concealment, and attrition/dropouts reporting, as well as the washout duration in crossover trials. These characteristics are presented in Supplementary Materials File S3.

## 3. Characteristics of Included Studies

### 3.1. Study Selection

**Figure 1:**
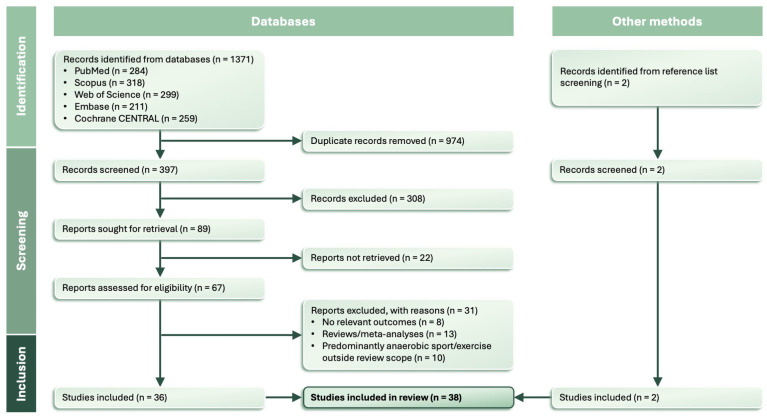
Flow diagram of the study selection process, showing identification of records through databases and reference-list screening.

Searches in PubMed, Scopus, Web of Science, Embase, and Cochrane CENTRAL identified 1371 records in total. After removal of 974 duplicates, 397 records were screened by title and abstract. Of these, 89 reports were sought for retrieval, and 67 full-text reports were assessed for eligibility. Thirty-one reports were excluded with reasons: no relevant outcomes (*n* = 8), reviews/meta-analyses (*n* = 13), and predominantly anaerobic sport/exercise outside the review scope (*n* = 10). Two additional records were identified through reference-list screening. Ultimately, a total of 38 RCTs, published between 1996 and 2025, were included in the scoping review (Figure 1).

### 3.2. Publication Years and Geographic Distribution

A total of 38 RCTs were included (Figure 1), published between 1996 and 2025. The largest numbers of publications originated from the USA (*n* = 6), Australia (*n* = 5), Brazil (*n* = 4), and the UK (*n* = 4). Studies were also conducted in other European countries, including Belgium, Poland, Finland, and Hungary, as well as in Asian countries such as Japan, Taiwan, and Iran.

### 3.3. Gender Distribution

The gender distribution of participants showed a clear predominance of men. Twenty-five studies included only male participants, whereas only one study analyzed women exclusively [11]. The remaining 12 studies included mixed-sex groups or both sexes in varying proportions, such as 19 men and 21 women in one study [12] and 7 men and 11 women in another [13]. Sample sizes ranged from 8 participants [14] to 40 participants [12]. This distribution limits sex-specific interpretation of the evidence, particularly for outcomes that may be influenced by sex-related physiological or hormonal factors.

### 3.4. Participant Training Status

The included studies varied substantially in participant training status. Several trials enrolled trained or competitive athletes, including swimmers, cyclists, runners, triathletes, rowers, canoeists/kayak paddlers, and team-sport athletes [11,15,16,17,18]. Some studies specifically involved elite or national-level athletes, whereas others included physically active, recreationally active, not regularly training, untrained, or adaptive-sport participants [12,19,20]. To reflect this heterogeneity, these participant categories were described separately, and findings were interpreted cautiously without assuming direct transferability across training-status groups.

### 3.5. Intervention Characteristics

Across trials, supplementation strategies were dominated by short-term loading protocols, most commonly consisting of a loading phase of 20 g/day for 5–7 days, often followed by a maintenance phase of 2–5 g/day. In the majority of studies (*n* = 27), approximately 20 g/day (or 0.3 g/kg body weight) was administered over 5–7 days. The shortest intervention lasted 5 days, whereas the longest protocols, which included a maintenance phase, extended to 4–10 weeks [21,22]. In some of the more recent studies, lower doses (3–6 g/day) administered over a longer period (33 days) without a loading phase were also reported [12].

### 3.6. Exercise Modalities and Disciplines

Supplementation was evaluated across a wide range of endurance and mixed-sport contexts, including sport-specific cohorts and broader exercise-based populations. Swimming was the most frequently studied discipline (*n* = 9 studies), followed by cycling-related cohorts (*n* = 7 studies) and rowing/canoeing/kayaking (*n* = 5 studies). The cycling-related category included studies of cyclists only as well as mixed cyclist–triathlete groups. The remaining studies involved runners, triathletes, recreationally active individuals, and, in one case, wheelchair athletes [23], as well as athletes from team, racket, and intermittent field/court sports. These latter sports, including soccer, basketball, baseball, tchoukball, ice hockey, and squash, were grouped as mixed-sport contexts rather than endurance disciplines.

### 3.7. Study Design

Parallel-group studies were the most common design in the analyzed dataset, accounting for 29 of the 38 included trials. This design was frequently used in longer-term supplementation interventions lasting 4–6 weeks [22,24,25]. The remaining studies used crossover designs, mainly in short-term protocols [14,15,16,19,23,26,27,28]. Reported washout periods in crossover trials generally ranged from 28 to 30 days.

## 4. Performance Effects of Creatine: Endurance Outcomes and Aerobic Capacity

### 4.1. Repeated-Sprint and High-Intensity Intermittent Tests

Creatine supplementation was most frequently associated with reported favorable changes in high-intensity intermittent performance, particularly in repeated sprint protocols, repeated cycling sprint tests, and power-endurance trials (Table 1). The outcomes for which favorable changes were most often reported were repeated-sprint ability, peak power output (PPO), total work done (TWD), and performance in short-duration, high-intensity tasks. In cyclists, Vandebuerie et al. (1998) [29] reported statistically significant between-group differences versus placebo for sprint peak power (+8.2%, *p* < 0.05) and sprint mean power (+9.29%, *p* < 0.05) after creatine loading. In soccer players, Deminice et al. (2013) [30] reported statistically significant between-group differences versus placebo for average power (+15.1%, *p* < 0.05), maximum power (+18.8%, *p* < 0.05), and minimum power (+26.1%, *p* < 0.05). However, the fatigue index showed only a non-significant percentage difference versus placebo. In swimmers, Dabidi Roshan et al. (2013) [31] reported a statistically significant between-group difference versus placebo for speed decrement measured 60 s after the third 50 m sprint (−60.2%, *p* < 0.05), whereas the corresponding value measured at 180 s was non-significant. Other studies reported favorable within-group changes from baseline, but not all were accompanied by statistically significant between-group differences versus placebo [16,17,24,25,32,33]. These findings were most often reported in protocols involving multiple bouts of high-intensity exercise with limited recovery, which is consistent with the proposed role of phosphocreatine-mediated ATP resynthesis during intermittent high-intensity exercise.

### 4.2. Rowing and Canoeing/Kayaking Performance Outcomes

Several studies in rowing and canoeing/kayaking reported favorable changes in short-duration, high-intensity performance outcomes, although these disciplines include both aerobic and anaerobic demands. For example, in elite rowers, creatine supplementation was associated with a statistically significant increase from baseline in time to exhaustion during anaerobic exercise (+19.6%, *p* < 0.01); however, the between-group difference versus placebo (+21.6%) was reported as a descriptive, non-significant percentage change [18]. In another rowing study, Lawrence et al. (1997) [42] reported small, non-significant percentage changes in 2500 m rowing ergometer performance time, both relative to baseline (−0.64%) and versus placebo (+0.96%). In kayak paddlers, between-group differences versus placebo in total work done expressed in kJ were +16.2% for the 90 s test and +6.63% for the 300 s test, both reported as statistically significant effects, whereas the +13.6% difference during the 150 s test was a non-significant percentage change [40]. Total work done expressed in W showed only small, non-significant percentage differences versus placebo across the 90, 150, and 300 s tests (Table 1) [40].

In canoeists, Wang et al. (2017) [34] reported a statistically significant within-group reduction from baseline in optimal individual post-activation potentiation time (−16.7%, *p* < 0.05), but no statistically significant between-group difference versus placebo was reported. This finding was therefore interpreted as a baseline change within the creatine group rather than as a placebo-superior effect.

### 4.3. Swimming and Sport-Specific Hydrodynamic Outcomes

In swimming, a study conducted exclusively in female athletes reported changes in hydrodynamic variables [11]. Swimming velocity showed only small, non-significant percentage changes from baseline (+0.68%) and versus placebo (+2.07%). Active drag force decreased significantly from baseline (−16.2%, *p* < 0.05), but the between-group difference versus placebo was +3.68% and was reported as statistically significant in the opposite direction (Table 1) [11]. The hydrodynamic coefficient showed a statistically significant reduction from baseline (−20.4%, *p* < 0.05), whereas the between-group difference versus placebo (−18.8%) was non-significant. Power output decreased significantly from baseline (−18.8%, *p* < 0.05), while the between-group difference versus placebo (+1.47%) was a non-significant percentage change. Overall, this study reported changes in selected swimming and hydrodynamic variables after creatine supplementation, but the direction, statistical support, and practical relevance of these findings were mixed. They should therefore be interpreted cautiously and not generalized beyond this specific study.

### 4.4. End-Phase Sprinting During Prolonged Exercise

Three studies reported favorable changes in end-phase sprinting capacity (“finishing kick”) or power output during prolonged exercise models [16,28,29]. In these studies, creatine supplementation was examined in exercise contexts that combined sustained effort with end-phase high-intensity efforts, such as sprint work performed after prolonged cycling. Reported changes were not uniform across all outcomes, but the findings suggest a possible role for creatine in tasks in which prolonged exercise is followed by short bursts of maximal or near-maximal intensity in the final phase of the task. These observations, however, should be interpreted cautiously because the number of studies was small and the protocols differed across trials.

### 4.5. Strength and Power Tests in Mixed-Sport Contexts

The data in Table 1 suggest that creatine supplementation may influence selected strength- and power-related parameters in mixed-sport contexts, although findings varied across tests and movement patterns. In intermittent team-sport athletes, including baseball, basketball, and tchoukball players, Wang et al. (2018) [25] reported a statistically significant increase from baseline in half-squat 1-RM strength (+33.4%, *p* < 0.05), with a descriptive placebo-relative difference of +7.65%. Other explosive-power outcomes were less consistent. Jump height increased by 19.7% from baseline, with a 1.24% difference relative to placebo, whereas PPO increased by 11.7% from baseline but showed an unfavorable placebo-relative comparison of −2.2%. These latter changes were not reported as statistically significant.

Findings for isolated muscle strength were also inconsistent. In ice hockey players, Cornish et al. (2006) [36] reported small placebo-relative changes in isokinetic knee-extension and knee-flexion outcomes, including +3.51% for knee-extension peak torque and −2.36% for knee-flexion peak torque. Average power changes were similarly small for knee extension (−0.6%) and knee flexion (+3.1%). These isokinetic outcomes were not reported as statistically significant [36]. Overall, these findings suggest that creatine may be associated with selected strength-related favorable changes in mixed-sport contexts, but the evidence remains outcome-specific and limited.

### 4.6. Aerobic Capacity, Time Trials, and Steady-State Outcomes

Time-trial and longer-duration performance outcomes, including 1 h cycling distance, 1 km cycling time, 2500 m rowing ergometer performance, and longer maximal-effort tests, were generally less consistent than repeated-sprint or short-duration high-intensity outcomes. In cyclists, Bellinger et al. (2000) [39] reported a 1.79% increase from baseline and a 1.53% difference relative to placebo in 1 h cycling trial distance, whereas De Andrade Nemezio et al. (2015) [35] reported no favorable pattern across 1 km cycling outcomes, including 1 km time, power output, power output relative to peak power output, and total work done. Similarly, Lawrence et al. (1997) [42] reported only small changes in 2500 m rowing ergometer performance time, with a −0.64% change from baseline and a +0.96% comparison relative to placebo.

The duration of effort appears to be an important factor in aerobic–anaerobic disciplines. In kayak paddlers, McNaughton et al. (1998) [40] reported placebo-relative increases in TWD of +16.2% during the 90 s test, +13.6% during the 150 s test, and +6.63% during the 300 s test. The 90 s and 300 s comparisons were reported as statistically significant, whereas the 150 s comparison was descriptive. The smaller placebo-relative change during the 300 s test than during the 90 s and 150 s tests suggests that the ergogenic effect of creatine may diminish as the contribution of phosphagen-dependent energy turnover becomes less dominant. Chwalbińska-Moneta (2003) [18] also reported an increase from baseline in time to exhaustion during all-out anaerobic exercise in rowers; however, the placebo-relative comparison was descriptive and was not reported as statistically significant. This outcome should therefore be interpreted as a high-intensity performance measure rather than direct evidence of improved steady-state aerobic capacity.

Collectively, the available evidence suggests that performance changes were more consistent in short-term, high-intensity, and intermittent tasks, whereas evidence for longer time-trial, steady-state, and endurance-dominant outcomes remains limited and equivocal.

## 5. Effects of Creatine on Biochemical Markers of Recovery and Exercise Stress

Across RCTs, creatine supplementation was associated with changes in several biochemical measures related to creatine and PCr availability, inflammatory responses, muscle damage, and broader exercise stress (Table 2). These circulating or intramuscular markers were interpreted as biochemical indicators that may be relevant to recovery-related processes, rather than as direct measures of functional recovery. Because biomarker sampling time points differed across studies, ranging from immediately post-exercise to 24–48 h after exercise or competition, CK, LDH, and inflammatory-marker responses were interpreted descriptively and with caution.

### 5.1. Muscle Creatine and Phosphocreatine Availability

Studies that directly assessed muscle metabolites using vastus lateralis biopsy samples reported changes in intramuscular creatine-related measures after short-term creatine loading, typically 20 g/day for 5–6 days [20,22,32,49]. Roberts et al. (2016) [20], in recreationally active participants, reported statistically significant increases in muscle phosphocreatine (PCr) both from baseline (+16.8%, *p* < 0.01) and versus placebo (+16.0%, *p* < 0.01) after 6 days of supplementation. Similarly, van Loon et al. (2003) [22], in untrained participants, reported statistically significant increases in muscle PCr and total creatine after the 5-day loading phase, although muscle PCr had returned to baseline by the latter maintenance-phase assessment (Table 2). Preen et al. (2001) [32], in cyclists, reported a statistically significant within-group increase (+12.5%, *p* < 0.05) from baseline in resting muscle PCr after 5 days of creatine supplementation; however, the difference versus placebo (+8.24%) was descriptive and was not reported as statistically significant. Finn et al. (2001) [49], in triathletes, reported elevated intramuscular stores of total and free creatine after creatine supplementation, whereas the increase in PCr did not reach statistical significance. Importantly, Finn et al. also found no ergogenic effect during four repeated 20 s all-out cycle sprints with 20 s recovery periods, suggesting that increased intramuscular creatine availability did not necessarily translate into improved performance under that specific repeated-sprint protocol.

### 5.2. Circulating Inflammatory Markers

Three included studies reported lower circulating inflammatory markers after creatine supplementation compared with placebo following strenuous exercise [30,46,48]. In trials assessing systemic inflammatory responses, short-term creatine loading, typically approximately 20 g/day or 0.3 g/kg/day for 5–7 days, was associated with statistically significant between-group differences versus placebo in selected inflammatory markers, including CRP, TNF-α, IFN-α, IL-1β, and PGE_2_ (Table 2). Bassit et al. (2008) [46] reported significantly lower plasma IFN-α, IL-1β, PGE_2_, and TNF-α concentrations versus placebo after a half-ironman competition, although IL-6 was not different between groups. Similarly, Deminice et al. (2013) [30] reported lower concentrations versus placebo for CRP (−32.5%, *p* < 0.01) and TNF-α (−23.8%, *p* < 0.05), with both differences reaching statistical significance, after repeated-sprint exercise in trained soccer players. Santos et al. (2004) [48] also reported significantly lower PGE_2_ (−60.9%, *p* < 0.05) and TNF-α (−33.7%, *p* < 0.05) concentrations versus placebo after a 30 km race. No included study reported inflammatory-marker findings based only on within-group baseline changes. Because relatively few studies assessed inflammatory markers and the exercise models differed, these findings should be interpreted cautiously.

### 5.3. Circulating Muscle Damage Markers

Evidence for circulating indirect markers of muscle damage, such as CK and LDH, was mixed across studies involving rowers, swimmers, runners, soccer players, and intermittent team-sport athletes (Table 2). Findings differed in the direction of the creatine effect, statistical support, and reporting format across studies [21,25,30,31,45,47,48]. In intermittent team-sport athletes, Wang et al. (2018) [25] reported significantly lower blood CK activity in the creatine group than in the placebo group after complex-training bouts, whereas Shi (2005) [47] reported significantly lower serum CK change scores following creatine supplementation versus placebo. These latter data, however, were reported as absolute change values rather than percentage changes. In contrast, Dabidi Roshan et al. (2013) [31] reported non-significant increases in plasma CK after repeated sprint-swimming bouts in both creatine and placebo groups, with no statistically significant between-group difference, even though the creatine group showed lower fatigue and improved performance. Atashak and Jafari (2012) [45] reported a statistically significant increase in serum CK activity in soccer players after one week of creatine supplementation, both from baseline (+174%, *p* < 0.05) and versus placebo (+109%, *p* < 0.05). Deminice et al. (2013) [30], also in soccer players, reported only a small descriptive increase in plasma CK from baseline (+6.79%), but the post-exercise CK value was significantly higher versus placebo (+33.8%, *p* < 0.05). Together, these two studies do not support a consistent CK-lowering effect of creatine in soccer players. In runners, Santos et al. (2004) [48] reported a lower plasma CK response versus placebo, but this comparison was not clearly reported as statistically significant. In rowers, Fernández-Landa et al. (2020) [21] reported only descriptive, non-significant percentage changes in CK from baseline and versus placebo. Thus, CK findings varied across exercise models, sampling approaches, and study contexts.

Reported LDH findings were similarly inconsistent. In soccer players, Atashak and Jafari (2012) [45] reported only modest descriptive increases in serum LDH from baseline (+9.0%) and versus placebo (+6.86%). Deminice et al. (2013) [30] also reported only small descriptive LDH changes in the creatine group from baseline (+2.43%) and versus placebo (+4.42%). In runners, Santos et al. (2004) [48] reported a lower plasma LDH response versus placebo after a 30 km race, and this comparison was reported as statistically significant. Fernández-Landa et al. (2020) [21] reported only descriptive, non-significant LDH changes in rowers, both from baseline and versus placebo.

Overall, CK and LDH responses did not show a consistent pattern across studies, and these circulating markers should be interpreted as indirect indicators of muscle damage rather than direct measures of structural muscle disruption.

## 6. Effects of Creatine Supplementation on Body Composition

Research on the effects of creatine supplementation on body mass and body composition was inconsistent across the included studies. The most frequently reported outcome was total body mass (TBM), whereas fat-free mass (FFM), total fat mass (TFM), percentage fat mass (PFM), and percentage body water (PBW) were assessed less consistently (Table 3). Therefore, body composition findings should be interpreted cautiously, particularly because the assessment methods differed between studies and varied in sensitivity to hydration-related changes.

TBM increased from baseline in most studies with available baseline data. Specifically, baseline increases were observed in 15 of the 19 trials reporting within-group TBM changes (Table 3), with six studies reporting statistically significant TBM increases [17,22,24,26,35,40]. Fernández-Landa et al. (2020) [21] reported a statistically significant decrease from baseline. Importantly, placebo-controlled comparisons were heterogeneous and did not show a consistent direction of effect. Thus, within-group increases in TBM after creatine loading should not be interpreted as consistent placebo-superior effects. Short-term loading protocols, typically 20 g/day for 5–7 days, were often associated with small increases in body mass, commonly in the range of approximately 0.6% to 2.5% [16,17,26,28,32,35,39,40,49]. These findings are consistent with the known tendency of creatine loading to increase body mass, but the available data do not allow firm conclusions regarding the functional relevance of this effect across endurance and mixed-sport contexts.

FFM was reported less frequently than TBM. Increases in FFM from baseline were reported in five trials [11,13,16,22,35], with statistically significant within-group increases reported in two studies [16,35]. However, placebo-controlled comparisons did not show a uniform pattern, and several placebo-controlled values were descriptive rather than statistically significant. Importantly, increases in FFM after short-term creatine supplementation should not be interpreted as direct evidence of meaningful skeletal muscle hypertrophy. Early changes may reflect increased intracellular water, fluid shifts, glycogen-associated water storage, or other hydration-related effects rather than accretion of contractile protein. This distinction is particularly important because the included studies used different body composition methods, including skinfolds, bioelectrical impedance analysis, and hydrodensitometry, which differ in their ability to distinguish tissue accretion from changes in hydration status.

Data on fat mass and body water were also limited. Among the four studies reporting placebo-relative changes in PFM, one reported a lower PFM value versus placebo [12], whereas three reported higher placebo-relative values [11,13,22]. These findings should be interpreted cautiously because small changes in estimated fat mass may reflect measurement variability or hydration-related shifts rather than true alterations in adiposity.

PBW was reported in only two studies, with divergent placebo-relative findings [11,13], further limiting interpretation of the mechanisms underlying changes in TBM and FFM. Increased muscle creatine concentrations may alter intracellular osmotic pressure and promote water retention, which could contribute to early increases in body mass [50]. However, the included studies do not allow this mechanism to be confirmed consistently across populations or methods.

Most interventions involved short-term loading protocols lasting 5–7 days, whereas relatively few studies examined longer supplementation periods. Longer interventions were less common and varied in design, including lower-dose protocols [13,28,40] and loading protocols followed by maintenance dosing [22,38]. These studies suggest that body mass may remain modestly elevated over several weeks in some contexts, but the pattern was not uniform and did not indicate progressive increases across all studies.

Taken together, body mass and body composition findings were heterogeneous, and the available evidence does not establish whether these changes translate into meaningful performance adaptations.

## 7. Mechanisms and Practical Implications

**Figure 2:**
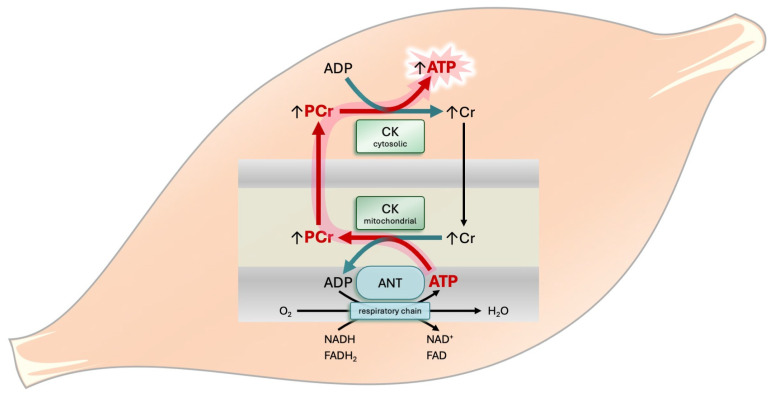
In skeletal muscle, the ATP–PCr (phosphagen) system rapidly resynthesizes ATP. Creatine supplementation can increase intramuscular phosphocreatine availability, supporting faster ATP resynthesis during brief, high-intensity efforts. ADP, adenosine diphosphate; ATP, adenosine triphosphate; ANT, adenine nucleotide translocator; CK, creatine kinase; Cr, creatine; PCr, phosphocreatine. Original figure created by the authors for this review.

The reported favorable changes in high-intensity and repeated-sprint performance are biologically plausible in light of creatine’s role in the PCr system (Figure 2). As summarized in Section 5.1, intramuscular creatine and PCr availability were not assessed consistently across all included studies; however, selected studies using muscle biopsy methods reported increases in muscle free creatine, PCr, or total creatine after short-term creatine loading [20,22,32,49]. These findings provide a mechanistic context for the performance outcomes, but they should not be interpreted as evidence that increased intramuscular creatine or PCr directly translated into improved performance across all protocols. Increased intramuscular creatine or PCr availability may support more rapid ATP resynthesis during short bursts of maximal or near-maximal effort, which could be relevant in tasks requiring repeated accelerations, sprint finishes, or brief high-power efforts after incomplete recovery. Accordingly, the performance findings were most apparent in repeated-sprint protocols, repeated cycling sprint tests, and power-endurance tasks, whereas evidence for steady-state aerobic outcomes was less consistent [13,30,32,40].

The present findings are broadly consistent with recent review-level evidence suggesting that creatine supplementation is more likely to benefit endurance or mixed-sport performance when the task includes intermittent high-intensity efforts, repeated accelerations, or end-spurts, rather than when performance is determined primarily by steady-state aerobic capacity [3]. At the same time, recent meta-analytic evidence in trained populations indicates that creatine monohydrate does not consistently improve endurance performance broadly, highlighting the need to distinguish between pure endurance outcomes and mixed or high-intensity performance components [8]. Therefore, the results of the present scoping review should not be interpreted as supporting creatine supplementation as a general endurance ergogenic aid. Rather, its potential appears context-dependent and may be greatest when endurance or mixed-sport performance includes high-intensity components.

Evidence for longer time-trial, steady-state, and endurance-dominant outcomes remains limited and equivocal. Reported effects on outcomes such as 1 h cycling distance, 1 km cycling time, 2500 m rowing ergometer performance, and longer maximal-effort tests were generally less consistent than those observed in short-duration or intermittent high-intensity tasks. Possible explanations include differences in test duration, participant training status, supplementation protocol, body mass changes, and the relative contribution of phosphagen-dependent energy turnover. Data on long-term adaptations, particularly in elite endurance and mixed-sport athletes, remain limited.

In practical terms, creatine supplementation may be most relevant for athletes whose sports or training sessions include repeated sprints, short recovery intervals, sprint finishes, high-power accelerations, or strength–power components. This may include selected contexts within cycling, rowing, canoeing/kayaking, swimming, and intermittent team or racket sports. However, practical recommendations should be individualized according to the sport, event duration, training phase, body mass considerations, and the specific performance outcome targeted. These contexts align with the proposed role of creatine in supporting rapid ATP resynthesis during repeated high-intensity efforts, surges, and end-spurts [3].

Creatine supplementation may influence selected recovery-related biochemical responses, but the available evidence remains limited and marker-specific. As summarized in Section 5.2, three studies reported lower concentrations of circulating inflammatory markers versus placebo after strenuous exercise, with these differences reaching statistical significance for CRP, TNF-α, IFN-α, IL-1β, and PGE_2_ [30,46,48]. These findings suggest that creatine may attenuate selected systemic inflammatory responses in some exercise contexts. However, because only a small number of studies assessed inflammatory outcomes, these findings should be interpreted cautiously. This caution is further supported by a recent systematic review and meta-analysis, which found that creatine supplementation did not consistently reduce systemic inflammatory biomarkers such as CRP or IL-6 across human populations, despite some favorable findings in endurance athletes exposed to intense physiological stress [51].

For circulating indirect markers of muscle damage, such as CK and LDH, the evidence was less consistent. As summarized in Section 5.3, CK and LDH responses varied across studies, with lower responses reported in some trials [25,47,48], higher CK values in others [30,45], and several null or descriptive findings [21,30,31,45,48]. For example, Dabidi Roshan et al. (2013) [31] reported improved intermittent sprint-swimming performance and reduced fatigue, but no statistically significant between-group difference in plasma CK after creatine supplementation. Overall, these findings suggest that recovery-related biochemical effects may occur in selected contexts, but they do not yet support a consistent or generalizable recovery benefit across endurance and mixed-sport populations.

Consistent with creatine supplementation protocols described in sport nutrition guidance [7], a typical creatine loading phase of approximately 20 g/day for 5–7 days, sometimes followed by maintenance dosing of 2–5 g/day, remained the most common approach across the included studies (Table 1, Table 2 and Table 3). Creatine was administered with carbohydrate in 19 studies and without carbohydrate in 19 studies. This distinction may be relevant because carbohydrate co-ingestion has been reported to enhance creatine retention, muscle creatine accumulation, and muscle glycogen content [7,20]. Roberts et al. (2016) [20] reported that creatine supplementation combined with a prescribed high-carbohydrate diet increased muscle glycogen resynthesis during recovery after prolonged exhaustive exercise. However, whether carbohydrate co-ingestion provides additional performance or recovery benefits beyond creatine alone remains uncertain. For example, Wang et al. (2026) [52] reported improvements in repeated-sprint mean power after creatine alone, creatine–carbohydrate, or creatine–carbohydrate–protein supplementation, but direct comparisons between supplementation groups were not statistically significant. Findings related to glycogen resynthesis and carbohydrate co-ingestion may therefore represent a relevant area for further study, but they should be interpreted cautiously because of the limited number of studies and differences in protocols, populations, and outcome measures.

From a body composition perspective, creatine supplementation was often associated with small increases in body mass or estimated FFM, particularly during short-term loading protocols (Table 3). However, these changes should not be interpreted as direct evidence of increased muscle contractile protein content. Early increases in body mass or FFM may partly reflect hydration-related changes, including increased intracellular water retention, rather than true contractile tissue accretion. Cell volumization has been proposed as a potential anabolic signal promoting protein synthesis [53], but the extent to which this mechanism contributes to meaningful training adaptations in endurance and mixed-sport contexts remains uncertain. Creatine-related training adaptations have been more clearly described in resistance-based settings [54], whereas evidence in endurance and mixed-sport contexts remains less direct and more context-dependent [30]. Changes in body mass may also have practical relevance in disciplines in which body mass influences performance, although this should be regarded as a theoretical and sport-specific consideration rather than a demonstrated impairment in the included trials. In weight-sensitive sports such as running, additional body mass could potentially increase the energetic cost of locomotion [3,55,56], whereas in swimming it may influence buoyancy or hydrodynamics. In contrast, in weight-neutral or power-oriented sports such as rowing or cycling, small increases in body mass may be less problematic, particularly when accompanied by improved high-intensity power output. Overall, the practical implications of creatine-associated body mass changes remain context-dependent and should be interpreted in relation to the sport, event duration, training phase, and athlete-specific goals.

## 8. Final Remarks

### 8.1. Limitations

Although the available evidence suggests potential ergogenic and physiological effects of creatine supplementation in selected endurance and mixed-sport contexts, several limitations should be acknowledged. First, as this was a scoping review, no formal risk-of-bias assessment or certainty-of-evidence grading was performed; therefore, the findings should be interpreted as a mapping and synthesis of the available literature rather than as definitive evidence for supplementation practice. Second, many included studies employed short-term interventions, typically lasting 5–7 days, which capture acute responses but do not fully reflect long-term adaptations during training cycles. Consequently, data on the chronic effects of creatine on endurance performance, metabolic flexibility, recovery, and injury resilience remain scarce. Third, the evidence base was characterized by small sample sizes and substantial heterogeneity in participant characteristics, training status, dosing regimens, exercise modalities, biomarker sampling time points, and outcome measures, which limited direct comparisons across studies and precluded robust quantitative synthesis. Some trials used multi-ingredient interventions, such as creatine combined with carbohydrates, which may have introduced synergistic or confounding effects and made it difficult to isolate the independent effect of creatine. Fourth, female-specific evidence remains very limited, as most included studies enrolled only male participants, whereas a single study included women exclusively, and mixed-sex studies generally included women in limited or variable proportions. This distribution substantially limits sex-specific interpretation of the evidence, particularly for outcomes that may be influenced by sex-related physiological or hormonal factors.

### 8.2. Future Research Directions

Future research should therefore aim to:

- Systematically evaluate sex-specific responses by including adequately powered cohorts of female participants, given known sex-related differences in muscle creatine content, hormonal milieu, and water balance.
- Investigate inter-individual variability and responder status by integrating molecular and genetic determinants of creatine uptake and storage.
- Use clearer sport classification systems that distinguish between steady-state endurance sports, intermittent endurance disciplines, mixed sports, combat sports, and team or racket sports with repeated high-intensity demands.
- Analyze and compare creatine-only interventions with combined supplementation protocols, particularly those involving carbohydrate co-ingestion, to better clarify the independent and additive effects of creatine.
- Standardize outcome reporting and assessment by using consistent definitions, measurement protocols, and sport-specific testing models for performance and body composition outcomes.
- Clarify the effects of creatine on inflammatory responses, circulating muscle damage markers, and recovery-related biochemical outcomes by using standardized protocols and consistent post-exercise sampling time points.
- Examine the long-term physiological and performance effects of creatine supplementation under real-world training conditions, particularly in trained and elite endurance or mixed-sport athletes.
- Assess the balance between potential ergogenic benefits and creatine-associated body mass gain in weight-sensitive disciplines and events to inform personalized supplementation strategies.

Overall, while creatine remains one of the most extensively studied ergogenic supplements, further research integrating performance, biochemical, and molecular endpoints is needed to clarify its long-term efficacy, mechanisms, and practical implications in endurance and mixed-sport contexts.

## 9. Conclusions

Creatine supplementation appears most promising in selected endurance and mixed-sport contexts, particularly when performance depends on repeated high-intensity efforts, sprint finishes, intermittent accelerations, or power-endurance demands. These findings are consistent with the proposed role of phosphocreatine resynthesis in supporting repeated bouts of high-intensity work. In contrast, evidence for steady-state aerobic capacity, recovery-related biomarkers, and body composition outcomes appears more variable and context-dependent.

Modest increases in body mass or estimated fat-free mass have been reported in some studies, but these changes should be interpreted cautiously because they may largely reflect water retention rather than contractile tissue accretion. Their practical relevance is likely sport-specific: small increases in mass may be less problematic in power-oriented or weight-neutral disciplines, such as rowing or cycling, but may require greater consideration in weight-sensitive sports such as running or swimming. Overall, current evidence is more consistent with a targeted, context-dependent role for creatine than with a universal benefit for endurance performance. Its greatest potential appears to lie in sports or training settings requiring rapid energy turnover, repeated intense efforts, or substantial between-bout recovery demands.

## Figures and Tables

**Table 1:** Effects of creatine supplementation on performance outcomes in randomized controlled trials.

Author, Year	Study Design	Intervention	Participants	Performance Outcomes
Yamaguchi et al., 2025 [12]	RCT DB	33 d, 3 g/d, *n* = 20 (10 F/10 M) vs. Plc, *n* = 20 (11 F/9 M)	not regularly training	ROM (deg): vs. Plc −0.24% MVC (kgf): vs. Plc +0.66% Upper arm circumference (cm): vs. Plc −0.39%
Meixner et al., 2025 [16]	RCT XO	5 d, 20 g/d, *n* = 25 (5 F/20 M)	cyclists	15 s work (J): vs. BL +2.72% *; vs. Plc +3.25%
Crisafulli et al., 2018 [24]	RCT DB	6 wk, 4 g/d, *n* = 12 (12 M) vs. Plc, *n* = 11 (11 M)	cyclists	PPO (W): vs. BL +4.16% *; vs. Plc +0.82%
Wang et al., 2018 [25]	RCT DB	6 d, 20 g/d, then 22 d, 2 g/d, + glucose, *n* = 15 (15 M) vs. Plc, *n* = 15 (15 M)	baseball, basketball, and tchoukball athletes	30 m sprint time (s): vs. BL −4.39% *; vs. Plc −1.19% Half squat 1-RM (kg): vs. BL +33.4% *; vs. Plc +7.65% Jump height (cm): vs. BL +19.7%; vs. Plc +1.24% PPO (W): vs. BL +11.7%; vs. Plc −2.2%
Dalton et al., 2017 [19]	RCT DB XO	6 d, 6 g/d, *n* = 28 (10 F/18 M)	recreationally active	Cycling time (s): vs. BL +1.85%; vs. Plc +0.78% Mean power (W): vs. BL +3.17%; vs. Plc +0.78%
Wang et al., 2017 [34]	RCT DB	6 d, 20 g/d, + glucose, *n* = 8 (8 M) vs. Plc, *n* = 9 (9 M)	canoeists	Optimal individual PAP time: vs. BL −16.7% *; vs. Plc NS
De Andrade Nemezio et al., 2015 [35]	RCT DB	5 d, 20 g/d, + glucose, *n* = 10 (10 M) vs. Plc, *n* = 9 (9 M)	cyclists	1 km time: vs. BL NS; vs. Plc +2.41% PO (W): vs. BL −0.13%; vs. Plc −3.11% PO relative to PPO: vs. BL +0.3%; vs. Plc −1.7% TWD over the trial (kJ): vs. BL +0.3%; vs. Plc −0.59%
Deminice et al., 2013 [30]	RCT DB	7 d, 0.3 g/kg/d, *n* = 13 (13 M) vs. Plc, *n* = 12 (12 M)	soccer players	Average power (W): vs. BL +17.1%; vs. Plc +15.1% * Maximum power (W): vs. BL +11.4%; vs. Plc +18.8% * Minimum power (W): vs. BL +40.3%; vs. Plc +26.1% * Fatigue index (W/s): vs. BL −26.3%; vs. Plc −2.33%
Dabidi Roshan et al., 2013 [31]	RCT	6 d, 20 g/d, *n* = 8 (8 M) vs. Plc, *n* = 8 (8 M)	swimmers	Speed decrement 60 s after the third 50 m sprint: vs. Plc −60.2% * Speed decrement 180 s after the third 50 m sprint: vs. Plc −17.3%
Juhász et al., 2009 [17]	RCT DB	5 d, 20 g/d, + glucose, *n* = 8 (8 M) vs. Plc, *n* = 8 (8 M)	swimmers	100 m fin swimming time, trial 1: vs. BL −3.61% *; vs. Plc −3.06% 100 m fin swimming time, trial 2: vs. BL −3.69% *; vs. Plc −3.21%
Branch et al., 2007 [28]	RCT DB XO	5 d, 20 g/d, *n* = 7 (7 M)	cyclists and triathletes	PO (W) first 50 min: vs. BL NS; vs. Plc +1.67% PO (W) last 10 min: vs. BL −0.56%; vs. Plc +2.3% TWD (kJ): vs. BL −0.08%; vs. Plc +1.8%
Silva et al., 2007 [11]	RCT DB	21 d, 20 g/d, + maltodextrin, *n* = 8 (8 F) vs. Plc, *n* = 8 (8 F)	swimmers	Swimming velocity MSV25 (m/s): vs. BL +0.68%; vs. Plc +2.07% Active drag force Df (N): vs. BL −16.2% *; vs. Plc +3.68% * Hydrodynamic coefficient Cx: vs. BL −20.4% *; vs. Plc −18.8% PO (W): vs. BL −18.8% *; vs. Plc +1.47%
Perret et al., 2006 [23]	RCT DB XO	6 d, 20 g/d, *n* = 6 (2 F/4 M)	wheelchair athletes	Max. velocity (km/h): vs. BL +0.63%; vs. Plc −3.32% Time for 800 m (s): vs. BL −2.24%; vs. Plc +1.01%
Cornish et al., 2006 [36]	RCT DB	5 d, 0.3 g/kg/d, + sucrose, *n* = 9 (9 M) vs. Plc, *n* = 8 (8 M)	ice-hockey players	Isokinetic average power (W) for knee extension at 60°: vs. BL +1.28%; vs. Plc −0.6% Isokinetic average power (W) for knee flexion at 60°: vs. BL +2.9%; vs. Plc +3.1% Isokinetic peak torque (Nm) for knee extension at 60°: vs. BL +1.59%; vs. Plc +3.51% Isokinetic peak torque (Nm) for knee flexion at 60°: vs. BL +1.58%; vs. Plc −2.36
Mendes et al., 2004 [37]	RCT DB	7 d, 20 g/d, + carbohydrates, *n* = 9 vs. Plc, *n* = 9 (6 F/12 M)	swimmers	50 m time: vs. BL +1.85%; vs. Plc −3.87% 100 m time: vs. BL +0.5%; vs. Plc +2.56%
Mero et al., 2004 [15]	RCT DB XO	6 d, 20 g/d, *n* = 16 (8 F/8 M)	swimmers	100 m sprint times: vs. BL +0.16%; vs. Plc −1.56%
van Loon et al., 2003 [22]	RCT DB	5 d, 20 g/d, then 37 d, 2 g/d, + glucose, *n* = 9 (9 M) vs. Plc, *n* = 10 (10 M)	untrained	PPO (W) day 6: vs. BL +7.3% PPO (W) day 42: vs. BL +6.47%
Chwalbińska-Moneta, 2003 [18]	RCT DB	5 d, 20 g/d, *n* = 8 (8 M) vs. Plc, *n* = 8 (8 M)	rowers	Time to exhaustion during all-out anaerobic exercise: vs. BL +19.6% **; vs. Plc +21.6%
van Schuylenbergh et al., 2003 [33]	RCT DB	7 d, 7 g/d, + maltodextrin, *n* = 7 (7 M) vs. Plc, *n* = 7 (7 M)	cyclists and triathletes	Sprint peak power (W): vs. BL +2.79% *; vs. Plc +14.0% Sprint mean power (W): vs. BL +3.67% *; vs. Plc +11.9%
Dawson et al., 2002 [38]	RCT SB	5 d, 20 g/d, then 22 d, 5 g/d, + glucose, *n* = 10 (5 F/5 M) vs. Plc, *n* = 10 (5 F/5 M)	swimmers	50 m time: vs. BL −1.53%; vs. Plc +0.5% 100 m time: vs. BL −2.93%; vs. Plc +0.66%
Romer et al., 2001 [26]	RCT DB XO	5 d, 20 g/d, *n* = 9 (1 F/8 M)	squash players	Mean set sprint time: vs. BL −4.74%; vs. Plc −2.84% *
Preen et al., 2001 [32]	RCT DB	5 d, 20 g/d, + glucose, *n* = 7 (7 M) vs. Plc, *n* = 7 (7 M)	cyclists	5 × 6 s (24 s recovery) work (kJ): vs. BL +5.01%; vs. Plc +4.65% 6 × 6 s (54 s recovery) work (kJ): vs. BL +6.2% *; vs. Plc +6.46% * 6 × 6 s (84 s recovery) work (kJ): vs. BL +6.86% *; vs. Plc +6.21% * TWD during the 80-min test (kJ): vs. BL +6.0% *; vs. Plc +5.75%
Bellinger et al., 2000 [39]	RCT DB	7 d, 20 g/d, *n* = 10 (10 M) vs. Plc, *n* = 10 (10 M)	cyclists	1 h cycling trial distance: vs. BL +1.79%; vs. Plc +1.53%
McNaughton et al., 1998 [40]	RCT XO	5 d, 20 g/d, + glucose, *n* = 8 (8 M) vs. Plc, *n* = 8 (8 M)	kayak paddlers	TWD (kJ) in 90 s: vs. Plc +16.2% ** TWD (kJ) in 150 s: vs. Plc +13.6% TWD (kJ) in 300 s: vs. Plc +6.63% * TWD (W) in 90 s: vs. Plc +0.68% TWD (W) in 150 s: vs. Plc +2.28% TWD (W) in 300 s: vs. Plc +2.31%
Peyrebrune et al., 1998 [41]	RCT DB	5 d, 9 g/d, + glucose and maltodextrin, *n* = 7 (7 M) vs. Plc, *n* = 7 (7 M)	swimmers	50-yard time (s): vs. BL +1.26%; vs. Plc −0.9%
Vanakoski et al., 1998 [14]	RCT DB XO	3 d, 0.3 g/kg/d, *n* = 7 (2 F/5 M)	runners	Anaerobic bout 1: vs. Plc +0.7% Anaerobic bout 2: vs. Plc NS Anaerobic bout 3: vs. Plc NS
Vandebuerie et al., 1998 [29]	RCT DB XO	5 d, 25 g/d, + maltodextrin, *n* = 12 (12 M)	cyclists	Sprint peak power (W): vs. Plc +8.2% * Sprint mean power (W): vs. Plc +9.29% * Fatigue (%): vs. Plc −9.57%
Lawrence et al., 1997 [42]	RCT DB	5 d, 3.6–6.4 g/d, *n* = 10 (5 F/5 M) vs. Plc, *n* = 10 (5 F/5 M)	rowers	2500 m rowing ergometer performance time: vs. BL −0.64%; vs. Plc +0.96%
Grindstaff et al., 1997 [13]	RCT DB	9 d, 21 g/d, + maltodextrin, *n* = 10 (6 F/4 M) vs. Plc, *n* = 10 (5 F/5 M)	swimmers	Heat 1: 50 m time: vs. BL +0.44%; vs. Plc −1.85% Heat 1: 100 m time: vs. BL −0.43%; vs. Plc −2.37% * Heat 2: 50 m time: vs. BL −0.76%; vs. Plc −3.56% * Heat 2: 100 m time: vs. BL −1.39%; vs. Plc −3.52% * Heat 3: 50 m time: vs. BL +0.25%; vs. Plc −4.2% * Heat 3: 100 m time: vs. BL −0.55%; vs. Plc −4.37% *
Terrillion et al., 1997 [43]	RCT DB	5 d, 20 g/d, + sucrose, *n* = 6 (6 M) vs. Plc, *n* = 6 (6 M)	runners	Trial 1 time: vs. BL +0.45%; vs. Plc NS Trial 2 time: vs. BL −1.73%; vs. Plc −0.28%
Burke et al., 1996 [44]	RCT DB	5 d, 20 g/d, + sucrose, *n* = 16 (7 F/9 M) vs. Plc, *n* = 16 (7 F/9 M)	swimmers	25 m sprint time: vs. BL +0.77%; vs. Plc +0.54 50 m sprint time: vs. BL +0.75%; vs. Plc +1.84% 100 m sprint time: vs. BL +0.66%; vs. Plc +2.5%

1-RM, one-repetition maximum; BL, baseline; DB, double-blind; MVC, maximal voluntary contraction; NS, no statistically significant difference; PAP, post-activation potentiation; Plc, placebo; PO, power output; PPO, peak power output; RCT, randomized controlled trial; ROM, range of motion; SB, single-blind; TWD, total work done; XO, crossover. Values in the “Performance Outcomes” column distinguish changes relative to baseline (“vs. BL”) from differences or comparisons relative to placebo (“vs. Plc”). Asterisks indicate statistically significant effects as reported in the original studies (* *p* < 0.05; ** *p* < 0.01). Percentage changes without an asterisk are descriptive values and should not be interpreted as statistically significant unless explicitly marked. “NS” indicates that the original study reported no statistically significant difference. Where both within-group and placebo comparisons are shown, these are presented separately to avoid conflating baseline changes with between-group effects. Table created by the authors based on data from the studies cited in each row (see “Author, Year” column).

**Table 2:** Effects of creatine supplementation on biochemical markers related to muscle energetic status, inflammation, and muscle damage in randomized controlled trials.

Author, Year	Study Design	Intervention	Participants	Muscle PCr (%)	Circulating Inflammatory Markers (%)	Circulating Muscle Damage Markers (%)
Fernández-Landa et al., 2020 [21]	RCT DB	10 wk, 0.04 g/kg/d, *n* = 7 (7 M) vs. Plc, *n* = 7 (7 M)	rowers	ND	ND	CK: vs. BL −7.47 CK: vs. Plc +18.2 LDH: vs. BL +8.76 LDH: vs. Plc +0.01
Wang et al., 2018 [25]	RCT DB	6 d, 20 g/d, then 22 d, 2 g/d, *n* = 15 (15 M) vs. Plc, *n* = 15 (15 M)	baseball, basketball, and tchoukball athletes	ND	ND	CK: vs. Plc −20.4 *
Roberts et al., 2016 [20]	RCT	6 d, 20 g/d, *n* = 7 (7 M) vs. Plc, *n* = 7 (7 M)	recreationally active	vs. BL +16.8 ** vs. Plc +16.0 **	ND	ND
Dabidi Roshan et al., 2013 [31]	RCT	6 d, 20 g/d, *n* = 8 (8 M) vs. Plc, *n* = 8 (8 M)	swimmers	ND	ND	CK: vs. BL +12.8 CK: vs. Plc 0.0
Deminice et al., 2013 [30]	RCT DB	7 d, 0.3 g/kg/d, *n* = 13 (13 M) vs. Plc, *n* = 12 (12 M)	soccer players	ND	CRP: vs. Plc −32.5 ** TNF-α: vs. Plc −23.8 *	CK: vs. BL +6.79 CK: vs. Plc +33.8 * LDH: vs. BL +2.43 LDH: vs. Plc +4.42
Atashak & Jafari, 2012 [45]	RCT DB	7 d, 20 g/d, + glucose, *n* = 9 (9 M) vs. Plc, *n* = 9 (9 M)	soccer players	ND	ND	CK: vs. BL +174 * CK: vs. Plc +109 * LDH: vs. BL +9.0 LDH: vs. Plc +6.86
Bassit et al., 2008 [46]	RCT DB	5 d, 20 g/d, *n* = 5 (5 M) vs. Plc, *n* = 6 (6 M)	triathletes	ND	IFN-α: vs. Plc −80.1 * IL-1β: vs. Plc −71 * IL-6: vs. Plc NS PGE_2_: vs. Plc −91 * TNF-α: vs. Plc −64 *	ND
Shi, 2005 [47]	RCT	7 d, 20 g/d, *n* = 5 (5 M) vs. Plc, *n* = 5 (5 M)	basketball players	ND	ND	CK: vs. Plc −96.9 * U/L ^†^
Santos et al., 2004 [48]	RCT DB	5 d, 20 g/d, + maltodextrin, *n* = 18 (18 M) vs. Plc, *n* = 16 (16 M)	runners	ND	PGE_2_: vs. Plc −60.9 * TNF-α: vs. Plc −33.7 *	CK: vs. Plc −19.8 LDH: vs. Plc −38.0 *
van Loon et al., 2003 [22]	RCT DB	5 d, 20 g/d, then 37 d, 2 g/d, *n* = 9 (9 M) vs. Plc, *n* = 10 (10 M)	untrained	vs. BL +20.9 * vs. Plc +30.3 * then vs. BL −0.51 vs. Plc +6.57	ND	ND
Finn et al., 2001 [49]	RCT DB	5 d, 20 g/d, *n* = 8 (8 M) vs. Plc, *n* = 8 (8 M)	triathletes	vs. BL +11.9 vs. Plc +13.4	ND	ND
Preen et al., 2001 [32]	RCT DB	5 d, 20 g/d, + glucose, *n* = 7 (7 M) vs. Plc, *n* = 7 (7 M)	cyclists	vs. BL +12.5 * vs. Plc +8.24	ND	ND

BL, baseline; CK, creatine kinase; CRP, C-reactive protein; DB, double-blind; IFN-α, interferon α; IL-1β, interleukin-1β; LDH, lactate dehydrogenase; ND, not determined; NS, no statistically significant difference; PCr, phosphocreatine; PGE_2_, prostaglandin E_2_; Plc, placebo; RCT, randomized controlled trial; TNF-α, tumor necrosis factor α. Values in the “Muscle PCr,” “Circulating Inflammatory Markers,” and “Circulating Muscle Damage Markers” columns distinguish changes relative to baseline (“vs. BL”) from differences or comparisons relative to placebo (“vs. Plc”). Biomarker sampling time points differed across studies and included immediate post-exercise, 24 h, and 48 h post-exercise or post-competition assessments; this timing heterogeneity should be considered when interpreting CK, LDH, and inflammatory-marker responses. Asterisks indicate statistically significant effects as reported in the original studies (* *p* < 0.05; ** *p* < 0.01). Percentage changes without an asterisk are descriptive values and should not be interpreted as statistically significant unless explicitly marked. “NS” indicates that the original study reported no statistically significant difference. Where both within-group and placebo comparisons are shown, these are presented separately to avoid conflating baseline changes with between-group effects. ^†^ For Shi (2005) [47], the CK value is presented as an absolute between-group difference in serum CK change scores because the original study reported change values rather than post-exercise concentrations. Table created by the authors based on data from the studies cited in each row (see “Author, Year” column).

**Table 3:** Effects of creatine supplementation on body composition in randomized controlled trials across different populations.

Author, Year	Study Design /Methods	Intervention	Participants	TBM (%)	FFM (%)	TFM (%)	PFM (%)	PBW (%)
Yamaguchi et al., 2025 [12]	RCT DB BIA	33 d, 3 g/d, *n* = 20 (10 F/10 M) vs. Plc, *n* = 20 (11 F/9 M)	not regularly training	vs. BL −0.34 vs. Plc −3.17	ND	ND	vs. BL −2.60 vs. Plc −14.2	ND
Meixner et al., 2025 [16]	RCT XO BIA	5 d, 20 g/d, *n* = 25 (5 F/20 M)	cyclists	vs. BL +0.55 vs. Plc +0.83	vs. BL +1.59 * vs. Plc +1.59	ND	ND	ND
Fernández-Landa et al., 2020 [21]	RCT DB Scale	10 wk, 0.04 g/kg/d, *n* = 7 (7 M) vs. Plc, *n* = 7 (7 M)	rowers	vs. BL −3.2 * vs. Plc −1.75	ND	ND	ND	ND
Crisafulli et al., 2018 [24]	RCT DB Scale	6 wk, 4 g/d, *n* = 12 (12 M) vs. Plc, *n* = 11 (11 M)	cyclists	vs. BL +2.23 * vs. Plc −1.34	ND	ND	ND	ND
De Andrade Nemezio et al., 2015 [35]	RCT DB Scale and Skinfolds	5 d, 20 g/d, + glucose, *n* = 10 (10 M) vs. Plc, *n* = 9 (9 M)	cyclists	vs. BL +1.14 * vs. Plc −3.15	vs. BL +1.13 * vs. Plc −0.63	ND	ND	ND
Juhász et al., 2009 [17]	RCT DB BIA	5 d, 20 g/d, + glucose, *n* = 8 (8 M) vs. Plc, *n* = 8 (8 M)	swimmers	vs. BL +1.7 *	ND	ND	ND	ND
Branch et al., 2007 [28]	RCT DB XO Scale	5 d, 20 g/d, *n* = 9 (9 M)	cyclists, triathletes	vs. BL +0.62 vs. Plc +0.12	ND	ND	ND	ND
Silva et al., 2007 [11]	RCT DB BIA	21 d, 20 g/d, + maltodextrin, *n* = 8 (8 F) vs. Plc, *n* = 8 (8 F)	swimmers	vs. BL −0.35 vs. Plc +10.9	vs. BL +3.76 vs. Plc +31.3	ND	vs. BL −3.58 vs. Plc +8.61	vs. BL −1.90 vs. Plc −2.66
Perret et al., 2006 [23]	RCT DB XO Scale	6 d, 20 g/d, *n* = 6 (2 F/4 M)	wheelchair athletes	vs. BL 0 vs. Plc +0.16	ND	ND	ND	ND
Cornish et al., 2006 [36]	RCT DB Scale	5 d, 0.3 g/kg/d, + sucrose, *n* = 9 (9 M) vs. Plc, *n* = 6 (6 M)	ice-hockey players	vs. BL +1.03 vs. Plc −5.19	ND	ND	ND	ND
van Loon et al., 2003 [22]	RCT DB Hydrodensitometry	5 d, 20 g/d, then 37 d, 2 g/d, + glucose, *n* = 9 (9 M) vs. Plc, *n* = 10 (10 M)	untrained	vs. BL +1.50 * vs. Plc −4.53	vs. BL +1.75 vs. Plc −4.90	vs. BL 0 vs. Plc −2.11	vs. BL −1.45 vs. Plc +4.62	ND
Dawson et al., 2002 [38]	RCT SB Scale and Skinfolds	5 d, 20 g/d, then 22 d, 5 g/d, + glucose, *n* = 10 (5 F/5 M) vs. Plc, *n* = 10 (5 F/5 M)	swimmers	vs. BL +0.31 vs. Plc −3.02	ND	ND	ND	ND
Romer et al., 2001 [26]	RCT DB XO Scale and Skinfolds	5 d, 20 g/d, *n* = 9 (1 F/8 M)	squash players	vs. BL +1.36 *	ND	ND	ND	ND
Finn et al., 2001 [49]	RCT DB Hydrodensitometry	5 d, 20 g/d, *n* = 8 (8 M) vs. Plc, *n* = 8 (8 M)	triathletes	vs. BL +1.08 vs. Plc −3.51	ND	ND	ND	ND
Preen et al., 2001 [32]	RCT DB Scale	5 d, 20 g/d, + glucose, *n* = 7 (7 M) vs. Plc, *n* = 7 (7 M)	cyclists	vs. BL +1.22	ND	ND	ND	ND
Bellinger et al., 2000 [39]	RCT DB Scale	7 d, 20 g/d, *n* = 10 (10 M) vs. Plc, *n* = 10 (10 M)	cyclists	vs. BL +1.08 vs. Plc 0	ND	ND	ND	ND
McNaughton et al., 1998 [40]	RCT XO Scale	5 d, 20 g/d, + glucose, *n* = 8 (8 M) vs. Plc, *n* = 8 (8 M)	kayak paddlers	vs. BL +2.52 * vs. Plc +2.38 *	ND	ND	ND	ND
Lawrence et al., 1997 [42]	RCT DB Scale	5 d, 3.6–6.4 g/d, *n* = 10 (5 F/5 M) vs. Plc, *n* = 10 (5 F/5 M)	rowers	vs. BL +0.26 vs. Plc −3.96	ND	ND	ND	ND
Grindstaff et al., 1997 [13]	RCT DB Scale and Skinfolds	9 d, 21 g/d, + maltodextrin, *n* = 10 (6 F/4 M) vs. Plc, *n* = 10 (5 F/5 M)	swimmers	vs. BL +0.81 vs. Plc +3.47	vs. BL +1.15 vs. Plc −0.38	vs. BL −2.02 vs. Plc +29.3	vs. BL −1.94 vs. Plc +16.9	vs. BL +2.02 vs. Plc +5.11

BIA, bioelectrical impedance analysis; BL, baseline; DB, double-blind; FFM, fat-free mass; ND, not determined; PBW, percentage body water; PFM, percentage fat mass; Plc, placebo; RCT, randomized controlled trial; SB, single-blind; TBM, total body mass; TFM, total fat mass; XO, crossover. Values in the “TBM,” FFM,” TFM,” PFM,” and “PBW” columns distinguish changes relative to baseline (“vs. BL”) from differences or comparisons relative to placebo (“vs. Plc”). Asterisks indicate statistically significant effects as reported in the original studies (* *p* < 0.05). Percentage changes without an asterisk are descriptive values and should not be interpreted as statistically significant unless explicitly marked. Where both within-group and placebo comparisons are shown, these are presented separately to avoid conflating baseline changes with between-group effects. Table created by the authors based on data from the studies cited in each row (see “Author, Year” column).

## Data Availability

No new data were created or analyzed in this study. Data sharing is not applicable to this article.

## Supplementary Materials

The following supporting information can be downloaded at: https://www.mdpi.com/article/10.3390/nu18111677/s1, File S1: PRISMA 2020 Checklist; File S2: Search Strategies; File S3: Descriptive Methodological Characteristics of Included Studies.

## Abbreviations

The following abbreviations are used in this manuscript:

ADP	Adenosine diphosphate
ANT	Adenine nucleotide translocator
ATP	Adenosine triphosphate
BIA	Bioelectrical impedance analysis
BL	Baseline
CK	Creatine kinase
Cr	Creatine
CRP	C-reactive protein
DB	Double-blind
FFM	Fat-free mass
IFN-α	Interferon α
IL-1β	Interleukin-1β
IL-6	Interleukin-6
LDH	Lactate dehydrogenase
MVC	Maximal voluntary contraction
ND	Not determined
NS	No significant difference
PCr	Phosphocreatine
PBW	Percentage body water
PFM	Percentage fat mass
PGE_2_	Prostaglandin E_2_
Plc	Placebo
PO	Power output
PPO	Peak power output
RCT	Randomized controlled trial
ROM	Range of motion
SB	Single-blind
TBM	Total body mass
TFM	Total fat mass
TNF-α	Tumor necrosis factor α
TWD	Total work done
XO	Crossover
