# Supplementary material for: Creatine Supplementation in Endurance and Mixed-Sport Contexts: A Scoping Review of Performance, Recovery, and Body Composition
Source: Nutrients. 2026 May 24;18(11):1677. doi: 10.3390/nu18111677 (PMC13258674; doi:10.3390/nu18111677)
Supplement: Supplementary file 1 [file nutrients-18-01677-s001.zip › File S2.pdf]

Supplementary File S2

Creatine Supplementation in Endurance and Mixed-Sport Contexts: A Scoping Review of Performance, Recovery, and Body Composition

Igor Wesołowski, Jacek Dzienisiewicz, Dorota Langa, Wiesław Ziółkowski, Joanna Karbowska, and Zdzislaw Kochan

Search Strategies

PubMed

("Athletic Performance"[MeSH Terms] OR "Endurance Training"[MeSH Terms] OR athlete*[Title/Abstract] OR athletics[Title/Abstract] OR endurance[Title/Abstract] OR aerobic[Title/Abstract] OR distance[Title/Abstract] OR runner*[Title/Abstract] OR marathon*[Title/Abstract] OR cyclist*[Title/Abstract] OR triathlete*[Title/Abstract] OR triathlon[Title/Abstract] OR rower*[Title/Abstract] OR rowing[Title/Abstract] OR swimmer*[Title/Abstract] OR swimming[Title/Abstract] OR mma[Title/Abstract] OR "mixed martial arts"[Title/Abstract] OR boxing[Title/Abstract] OR "boxer*"[Title/Abstract]) AND (Creatine[Supplementary Concept] OR Creatine[MeSH Terms] OR "creatine monohydrate"[Title/Abstract] OR CrM[Title/Abstract] OR "creatine supplement*"[Title/Abstract] OR "creatine load*"[Title/Abstract] OR "creatine ethyl ester"[Title/Abstract] OR "kre-alkalyn"[Title/Abstract] OR creapure[Title/Abstract] OR Creatine*[Title/Abstract]) AND (strength[Title/Abstract] OR "muscle strength"[MeSH Terms] OR recovery[Title/Abstract] OR "muscle damage"[Title/Abstract] OR soreness[Title/Abstract] OR CRP[Title/Abstract] OR "serum CK"[Title/Abstract] OR "C-Reactive Protein"[Title/Abstract] OR "creatine kinase"[Title/Abstract] OR "lactate dehydrogenase"[Title/Abstract] OR LDH[Title/Abstract] OR lactate[Title/Abstract] OR "Interleukin-6"[Title/Abstract] OR "IL-6"[Title/Abstract]) AND "humans"[MeSH Terms] AND ("1996"[Date - Publication] : "2025"[Date - Publication]) AND placebo[Title/Abstract]

Scopus

TITLE-ABS-KEY("athletic performance" OR "endurance training" OR athlete* OR athletics OR endurance OR aerobic OR distance OR runner* OR marathon* OR cyclist* OR triathlete* OR triathlon OR rower* OR rowing OR swimmer* OR swimming OR mma OR "mixed martial arts" OR "mixed martial art" OR boxing OR boxer*) AND TITLE-ABS-KEY(creatine OR "creatine monohydrate" OR CrM OR "creatine supplement*" OR "creatine load*" OR "creatine ethyl ester" OR "kre-alkalyn" OR creapure OR creatine*) AND TITLE-ABS-KEY(strength OR "muscle strength" OR recovery OR "muscle damage" OR soreness OR CRP OR "serum CK" OR "creatine kinase" OR "C-reactive protein" OR "C reactive protein" OR "lactate dehydrogenase" OR LDH OR lactate OR "interleukin-6" OR "interleukin 6" OR "IL-6" OR "IL 6") AND TITLE-ABS-KEY(placebo OR placebo-controlled OR "placebo controlled") AND PUBYEAR > 1995 AND PUBYEAR < 2026

Web of Science

TS=("athletic performance" OR "endurance training" OR athlete* OR athletics OR endurance OR aerobic OR distance OR runner* OR marathon* OR cyclist* OR triathlete* OR triathlon OR rower* OR rowing OR swimmer* OR swimming OR mma OR "mixed martial arts" OR "mixed martial art" OR boxing OR boxer*) AND TS=(creatine OR "creatine monohydrate" OR CrM OR "creatine supplement*" OR "creatine load*" OR "creatine ethyl ester" OR "kre-alkalyn" OR creapure OR creatine*) AND TS=(strength OR "muscle strength" OR recovery OR "muscle damage" OR soreness OR CRP OR "serum CK" OR "creatine kinase" OR "C-reactive protein" OR "C reactive protein" OR "lactate dehydrogenase" OR LDH OR lactate OR "interleukin-6" OR "interleukin 6" OR "IL-6" OR "IL 6") AND TS=(placebo OR placebo-controlled OR "placebo controlled") AND PY=(1996-2025)

Embase

('athletic performance'/exp OR 'endurance training'/exp OR 'athlete'/exp OR athlete*:ti,ab,kw OR athletics:ti,ab,kw OR endurance:ti,ab,kw OR aerobic:ti,ab,kw OR distance:ti,ab,kw OR runner*:ti,ab,kw OR marathon*:ti,ab,kw OR cyclist*:ti,ab,kw OR triathlete*:ti,ab,kw OR triathlon:ti,ab,kw OR rower*:ti,ab,kw OR rowing:ti,ab,kw OR swimmer*:ti,ab,kw OR swimming:ti,ab,kw OR mma:ti,ab,kw OR 'mixed martial arts':ti,ab,kw OR 'mixed martial art':ti,ab,kw OR boxing:ti,ab,kw OR boxer*:ti,ab,kw) AND ('creatine'/exp OR 'creatine monohydrate':ti,ab,kw OR crm:ti,ab,kw OR 'creatine supplement*':ti,ab,kw OR 'creatine load*':ti,ab,kw OR 'creatine ethyl ester':ti,ab,kw OR 'kre-alkalyn':ti,ab,kw OR creapure:ti,ab,kw OR creatine*:ti,ab,kw) AND ('muscle strength'/exp OR strength:ti,ab,kw OR 'muscle strength':ti,ab,kw OR recovery:ti,ab,kw OR 'muscle damage':ti,ab,kw OR soreness:ti,ab,kw OR crp:ti,ab,kw OR 'serum ck':ti,ab,kw OR 'C reactive protein'/exp OR 'C reactive protein':ti,ab,kw OR 'C-reactive protein':ti,ab,kw OR 'creatine kinase'/exp OR 'creatine kinase':ti,ab,kw OR 'lactate dehydrogenase'/exp OR 'lactate dehydrogenase':ti,ab,kw OR ldh:ti,ab,kw OR lactate:ti,ab,kw OR 'interleukin 6'/exp OR 'interleukin 6':ti,ab,kw OR 'interleukin-6':ti,ab,kw OR 'IL 6':ti,ab,kw OR 'IL-6':ti,ab,kw) AND (placebo*:ti,ab,kw OR 'placebo controlled study'/exp OR 'placebo controlled':ti,ab,kw OR 'placebo-controlled':ti,ab,kw) AND 'human'/exp AND [1996-2025]/py

Cochrane CENTRAL

[mh "Athletic Performance"] OR [mh "Endurance Training"] OR athlete*:ti,ab,kw OR athletics:ti,ab,kw OR endurance:ti,ab,kw OR aerobic:ti,ab,kw OR distance:ti,ab,kw OR runner*:ti,ab,kw OR marathon*:ti,ab,kw OR cyclist*:ti,ab,kw OR triathlete*:ti,ab,kw OR triathlon:ti,ab,kw OR rower*:ti,ab,kw OR rowing:ti,ab,kw OR swimmer*:ti,ab,kw OR swimming:ti,ab,kw OR mma:ti,ab,kw OR "mixed martial arts":ti,ab,kw OR "mixed martial art":ti,ab,kw OR boxing:ti,ab,kw OR boxer*:ti,ab,kw
[mh Creatine] OR creatine*:ti,ab,kw OR "creatine monohydrate":ti,ab,kw OR CrM:ti,ab,kw OR "creatine supplement":ti,ab,kw OR "creatine supplements":ti,ab,kw OR "creatine supplementation":ti,ab,kw OR "creatine loading":ti,ab,kw OR "creatine load":ti,ab,kw OR "creatine ethyl ester":ti,ab,kw OR "kre-alkalyn":ti,ab,kw OR creapure:ti,ab,kw
[mh "Muscle Strength"] OR strength:ti,ab,kw OR "muscle strength":ti,ab,kw OR recovery:ti,ab,kw OR "muscle damage":ti,ab,kw OR soreness:ti,ab,kw OR CRP:ti,ab,kw OR "serum CK":ti,ab,kw OR [mh "C-Reactive Protein"] OR "C-reactive protein":ti,ab,kw OR "C reactive protein":ti,ab,kw OR [mh "Creatine Kinase"] OR "creatine kinase":ti,ab,kw OR [mh "Lactate Dehydrogenases"] OR "lactate dehydrogenase":ti,ab,kw OR LDH:ti,ab,kw OR lactate:ti,ab,kw OR [mh "Interleukin-6"] OR "interleukin-6":ti,ab,kw OR "interleukin 6":ti,ab,kw OR "IL-6":ti,ab,kw OR "IL 6":ti,ab,kw
placebo*:ti,ab,kw OR "placebo controlled":ti,ab,kw OR "placebo-controlled":ti,ab,kw
#1 AND #2 AND #3 AND #4

SPORTDiscus

(TI ("athletic performance" OR "endurance training" OR athlete* OR athletics OR endurance OR aerobic OR distance OR runner* OR marathon* OR cyclist* OR triathlete* OR triathlon OR rower* OR rowing OR swimmer* OR swimming OR mma OR "mixed martial arts" OR "mixed martial art" OR boxing OR boxer*) OR AB ("athletic performance" OR "endurance training" OR athlete* OR athletics OR endurance OR aerobic OR distance OR runner* OR marathon* OR cyclist* OR triathlete* OR triathlon OR rower* OR rowing OR swimmer* OR swimming OR mma OR "mixed martial arts" OR "mixed martial art" OR boxing OR boxer*) OR SU ("athletic performance" OR "endurance training" OR athletes OR athletics OR endurance OR aerobic exercise OR running OR marathon running OR cycling OR triathlon OR rowing OR swimming OR mixed martial arts OR boxing)) AND (TI (creatine OR "creatine monohydrate" OR CrM OR "creatine supplement*" OR "creatine load*" OR "creatine ethyl ester" OR "kre-alkalyn" OR creapure OR creatine*) OR AB (creatine OR "creatine monohydrate" OR CrM OR "creatine supplement*" OR "creatine load*" OR "creatine ethyl ester" OR "kre-alkalyn" OR creapure OR creatine*) OR SU (creatine OR "creatine supplementation" OR "dietary supplements" OR supplementation)) AND (TI (strength OR "muscle strength" OR recovery OR "muscle damage" OR soreness OR CRP OR "serum CK" OR "C-reactive protein" OR "C reactive protein" OR "creatine kinase" OR "lactate dehydrogenase" OR LDH OR lactate OR "interleukin-6" OR "interleukin 6" OR "IL-6" OR "IL 6") OR AB (strength OR "muscle strength" OR recovery OR "muscle damage" OR soreness OR CRP OR "serum CK" OR "C-reactive protein" OR "C reactive protein" OR "creatine kinase" OR "lactate dehydrogenase" OR LDH OR lactate OR "interleukin-6" OR "interleukin 6" OR "IL-6" OR "IL 6") OR SU ("muscle strength" OR recovery OR "muscle damage" OR soreness OR inflammation OR "creatine kinase"

OR lactate OR "lactate dehydrogenase")) AND (TI (placebo OR placebo*) OR AB (placebo OR placebo*) OR SU placebo)
