# Supplementary material for: Creatine Supplementation in Endurance and Mixed-Sport Contexts: A Scoping Review of Performance, Recovery, and Body Composition
Source: Nutrients. 2026 May 24;18(11):1677. doi: 10.3390/nu18111677 (PMC13258674; doi:10.3390/nu18111677)
Supplement: Supplementary file 1 [file nutrients-18-01677-s001.zip › File S3.pdf]

*Supplementary File S3*

# Creatine Supplementation in Endurance and Mixed-Sport Contexts: A Scoping Review of Performance, Recovery, and Body Composition

**Igor Wesołowski, Jacek Dzienisiewicz, Dorota Langa, Wiesław Ziółkowski, Joanna Karbowska, and Zdzislaw Kochan**

## Descriptive Methodological Characteristics of Included Studies

| Study | Randomization reported | Blinding | Allocation concealment reported | Attrition/dropouts reported | Washout adequacy for crossover studies |
|---|---|---|---|---|---|
| Yamaguchi et al., 2025 [12] | Yes; participants were randomly assigned to creatine or placebo, stratified by age and maximal muscle strength | Double-blind | Not reported | Yes; withdrawals/exclusions reported, with 40 participants included in the final analysis | Not applicable; parallel-group trial |
| Meixner et al., 2025 [16] | No; nonrandomized sequential crossover design | Participants blinded only | Not reported | Yes; 5 participants could not finish all required trials and 25 were included in the analysis | No washout between placebo and creatine conditions; authors state this was due to the relatively long washout period of creatine |
| Fernández-Landa et al., 2020 [21] | Yes; randomized to four groups by an independent statistician using a stratified block design | Double-blind | Not reported | Yes; no dropouts reported | Not applicable; parallel-group trial |
| Crisafulli et al., 2018 [24] | Yes; participants were randomly assigned to creatine-electrolyte or placebo groups | Double-blind | Not reported | Yes; 2 participants dropped out, 1 from each group | Not applicable; parallel-group trial |
| Wang et al., 2018 [25] | Yes; double-blind randomized matched-pair assignment to creatine or placebo groups | Double-blind | Not reported | Not reported; n = 15 per group reported throughout | Not applicable; parallel-group trial |
| Dalton et al., 2017 [19] | Yes; randomized, counterbalanced crossover design | Double-blind | Not reported | Yes; 6 participants did not complete the study; 28 were analyzed | Yes; 7-day washout between treatment periods |
| Wang et al., 2017 [34] | Yes; randomized assignment to creatine or placebo groups | Double-blind | Not reported | Not reported; 17 participants included and analyzed | Not applicable; parallel-group trial |
| Roberts et al., 2016 [20] | Yes; participants were randomly assigned to creatine or placebo groups | Not reported | Not reported | Not reported; 14 participants completed study procedures as reported | Not applicable; parallel-group trial |

| | | | | | |
|---|---|---|---|---|---|
| De Andrade Nemezio et al., 2015 [35] | Yes; participants were randomly allocated to creatine or placebo groups | Double-blind | Not reported | Yes; 5 participants did not complete all trials and were excluded from analysis | Not applicable; parallel-group trial |
| Dabidi Roshan et al., 2013 [31] | Yes; swimmers were randomly assigned to creatine or placebo groups | Not reported | Not reported | Not reported; 16 participants reported, 8 per group | Not applicable; parallel-group trial |
| Deminice et al., 2013 [30] | Yes; participants were randomly assigned to creatine or placebo groups | Double-blind | Not reported | Not reported; 25 participants reported, with 13 in creatine and 12 in placebo groups | Not applicable; parallel-group trial |
| Atashak & Jafari, 2012 [45] | Yes; participants were randomized into creatine and placebo groups | Double-blind | Not reported | Not reported; 18 participants reported, 9 per group | Not applicable; parallel-group trial |
| Juhász et al., 2009 [17] | Yes; participants were randomly assigned to creatine or placebo groups | Double-blind | Not reported | Yes; no side effects reported, but dropouts/attrition not explicitly reported | Not applicable; parallel-group trial |
| Bassit et al., 2008 [46] | Yes; athletes were randomly divided into creatine and control/placebo groups | Double-blind | Not reported | Not reported; 11 athletes reported, with 5 in creatine and 6 in control/placebo groups | Not applicable; parallel-group trial |
| Branch et al., 2007 [28] | Yes; randomized, counterbalanced crossover design | Double-blind | Not reported | Not reported; 7 participants completed all conditions as reported | Yes; 28-day washout between creatine and placebo conditions |
| Silva et al., 2007 [11] | Yes; participants were randomly assigned to creatine or placebo groups | Double-blind | Not reported | Not reported; 16 participants reported, 8 per group | Not applicable; parallel-group trial |
| Perret et al., 2006 [23] | Yes; randomized crossover design | Double-blind | Not reported | Not reported; 6 participants completed both treatment conditions as reported | Yes; ≥28-day / 4-week washout between supplementation periods |
| Cornish et al., 2006 [36] | Yes; participants were randomized to creatine or placebo groups | Double-blind | Not reported | Yes; 3 participants did not complete post-supplementation skating testing, and 2 did not complete post-supplementation knee extension/flexion testing | Not applicable; parallel-group trial |
| Shi, 2005 [47] | Not clearly reported; 20 athletes were divided into four groups using an orthogonal L4 design | Not reported | Not reported | Not reported; 20 participants reported, 5 per group | Not applicable; parallel-group trial |
| Mendes et al., 2004 [37] | Yes; swimmers were randomly divided | Double-blind | Not reported | Not reported; 18 participants reported, 9 per group | Not applicable; parallel-group trial |

| | | | | | |
|---|---|---|---|---|---|
| | into creatine or placebo groups | | | | |
| Mero et al., 2004 [15] | Yes; randomized, balanced crossover order | Double-blind | Not reported | Not reported; 16 participants completed both treatments as reported | Yes; ≥30-day washout between treatments |
| Santos et al., 2004 [48] | Not reported | Double-blind | Not reported | Not reported; 34 participants reported, with 18 in creatine and 16 in placebo/control groups | Not applicable; parallel-group trial |
| van Loon et al., 2003 [22] | Not clearly reported; subjects were group-matched and assigned to creatine or placebo groups | Double-blind | Not reported | Yes; one creatine-group participant was classified as a non-responder and excluded from statistical analyses | Not applicable; parallel-group trial |
| Chwalbińska-Moneta, 2003 [18] | Yes; participants were randomly assigned to creatine or placebo groups | Double-blind | Not reported | Not reported; 16 participants reported, 8 per group | Not applicable; parallel-group trial |
| van Schuylenbergh et al., 2003 [33] | Yes; participants were randomly assigned to creatine-pyruvate or placebo groups | Double-blind | Not reported | Not reported; 14 participants reported, 7 per group | Not applicable; parallel-group trial |
| Dawson et al., 2002 [38] | Yes; matched pairs by gender and 50-m swim time were randomly assigned to creatine or placebo groups | Single-blind | Not reported | Not reported; 20 participants reported, 10 per group | Not applicable; parallel-group trial |
| Romer et al., 2001 [26] | Yes; randomized, counterbalanced crossover design | Double-blind | Not reported | Yes; 1 participant was removed from analyses for non-adherence to preparation guidelines | Yes; 4-week washout between treatments |
| Finn et al., 2001 [49] | Yes; matched pairs were randomly allocated to creatine or placebo groups | Double-blind | Not reported | Not reported; 16 participants reported, with 8 per group | Not applicable; parallel-group trial |
| Preen et al., 2001 [32] | Not reported | Double-blind | Not reported | Not reported; 14 participants reported, 7 per group | Not applicable; parallel-group trial |
| Bellinger et al., 2000 [39] | Yes; creatine and placebo packages were randomly assigned numbers and then given to participants | Double-blind | Not reported | Not reported; 20 participants reported, 10 per group | Not applicable; parallel-group trial |
| McNaughton et al., 1998 [40] | Yes; participants were randomly assigned to creatine or placebo first, then crossed over | Not reported | Not reported | Not reported; 16 participants completed the repeated testing protocol as reported | Yes; 4-week washout between treatment periods |

| | | | | | |
|---|---|---|---|---|---|
| Peyrebrune et al., 1998 [41] | Yes; participants were randomly assigned to creatine or control/placebo groups | Double-blind | Not reported | Not reported; 14 participants reported, 7 per group | Not applicable; parallel-group trial |
| Vanakoski et al., 1998 [14] | Yes; randomized, balanced crossover design | Double-blind | Not reported | Yes; 8 participants enrolled, but 1 was excluded from analysis because of poor compliance with exercise protocols | Yes; ≥1-week washout between treatment sessions |
| Vandebuerie et al., 1998 [29] | Yes; randomized-order crossover design | Double-blind | Not reported | Yes; 1 participant was withdrawn because of severe diarrhoea and was excluded from analyses | Yes; 5-week washout between experimental conditions |
| Lawrence et al., 1997 [42] | Not clearly reported; participants were matched by sex and body mass, with one member of each pair assigned to creatine and the other to placebo | Double-blind | Not reported | Not reported; 20 participants reported, 10 per group | Not applicable; parallel-group trial |
| Grindstaff et al., 1997 [13] | Yes; participants were randomly assigned to creatine or placebo groups | Double-blind | Not reported | Not reported; 18 participants reported, 9 per group | Not applicable; parallel-group trial |
| Terrillion et al., 1997 [43] | Not clearly reported; matched-pair allocation to creatine or placebo groups | Double-blind | Not reported | Not reported; 12 participants reported, 6 per group | Not applicable; parallel-group trial |
| Burke et al., 1996 [44] | Yes; matched groups were randomly assigned to creatine or placebo | Double-blind | Not reported | Not reported; 32 participants reported, 16 per group | Not applicable; parallel-group trial |
